# Integral Betti signatures of brain, climate and financial networks compared to hyperbolic, Euclidean and spherical models

**DOI:** 10.1038/s41598-025-31700-z

**Published:** 2025-12-23

**Authors:** Luigi Caputi, Anna Pidnebesna, Jaroslav Hlinka

**Affiliations:** 1 Institute of Computer Science of the Czech Academy of Sciences, Pod Vodárenskou věží 271/2, 182 07 Prague, Czech Republic; 2 Present Address: Department of Mathematics, University of Bologna, Bologna, Italy; 3 National Institute of Mental Health, Topolová 748, 250 67 Klecany, Czech Republic

**Keywords:** Data manifolds, Functional connectivity, TDA, Betti curves, Hyperbolic geometry, Integral Betti signatures, Applied mathematics, Scientific data, Computational science

## Abstract

This paper extends the possibility to examine the underlying curvature of data through the lens of topology by using the Betti curves, tools of Persistent Homology. We show that low-dimensional Betti curve approximations effectively distinguish not only Euclidean, but also spherical and hyperbolic geometric matrices, both from purely random matrices as well as among themselves. We proved this by analysing the behaviour of Betti curves for various geometric matrices – i.e distance matrices of points randomly distributed on manifolds given by the Euclidean space, the sphere, and the hyperbolic space. We further show that the standard approach to network construction gives rise to (spurious) spherical geometry, and document the role of sample size and dimension to assess real-world connectivity matrices. Finally, we observe that real-world datasets coming from neuroscience, finance and climate seem to exhibit a hyperbolic character. The potential confounding “hyperbologenic effect” of intrinsic low-rank modular structures is evaluated.

## Introduction

The scientific understanding of phenomena around us depends on devising a formal model of reality, which is iteratively tested and further developed based on comparison with observations, and which, ultimately, provides prediction of further phenomena. Many real-world systems, such as the human brain or the Earth’s climate, fall within the category of complex systems, with structure neither random nor fully ordered. While the particular structure of such systems may differ from realization to realization, and may also dynamically change in time, the key challenge is to understand the organizing principles that gave rise to the structure, that is, to build a generative model.

Most often, real-world systems are of very high dimension, and thus, even after some initial dimensionality reduction, they call for representations in high dimensional spaces. Working with such high dimensional objects, although being possible, is often problematic both due to the computational demands, and to the limitations of reliability of such fine-grained analysis. Thus, additional low dimensionality reduction algorithms are usually employed. There is a strong and commonly accepted intuition that many real-world, high-dimensional, datasets have a lower dimensional representation; this is an assumption, usually referred to as the “manifold hypothesis”: *data in the form of point clouds in *$\mathbb {R}^n$* are sampled from (or, essentially, close to) a manifold *$M^d$* whose dimension d is smaller than the ambient dimension n*. The manifold *M* is called *data manifold*, and the recent need of new methodologies for analysing high dimensional data based on this hypothesis gave birth to a new research field, known as “manifold learning”. Experiments show that the manifold hypothesis is true for many real world datasets, and algorithms to test it have been introduced^1^. For instance, the space of natural images is surprisingly well approximated by a two-dimensional representation homeomorphic to the (2-dimensional) Klein bottle^2^; showing that the underlying topology of data manifolds can be quite unintuitive. Estimating and understanding the intrinsic geometrical and topological properties of data manifolds is important not just from a theoretical point of view, but also for extracting features and qualitative information from the data itself; e.g. in developing faster dimensionality-reduction algorithms.

The advantage of the manifold hypothesis is that it allows us to exploit additional theoretical structures and geometric properties of manifolds. Among others, Riemannian geometry is recently gaining much attention^3^. The usual linear tools, as Principal Component Analysis and Factor Analysis, work well when the data lies close to a linear, flat, subspace of $\mathbb {R}^n$. However, such linear methods do not work equally well when the data lies near a more complicated, nonlinear, manifold, and may fail to recover the intrinsic structure, along with related relevant information. Driven by these considerations, numerous nonlinear manifold learning methods and algorithms have been recently proposed; see, e.g.^4^ and the references therein. The notion of (Riemannian) *curvature* gives an intrinsic, strong, geometric, description of a (data) manifold and has led to the so-called Riemannian Manifold Learning^5^.

We refrain here from giving a technical account of techniques in Riemannian Manifold Learning. The main driving idea is the belief that data might actually be governed by a non-Euclidean, curved, geometry, rather than the most conventional, Euclidean, one. Developing non-Euclidean methods is therefore of great importance in testing and dealing with such frameworks. However, dealing with general manifolds of arbitrary curvature is computationally very difficult. Therefore, in this work, we shall focus on (highly symmetric) geometric models of constant curvature. It is well-known that Riemannian manifolds of constant (sectional) curvature, in any dimension *d*, are classified in three classes: of positive, vanishing, and negative curvature. Models of these spaces are the classical sphere $\mathbb {S}^d$, the Euclidean space $\mathbb {R}^d$, and the hyperbolic space $\mathbb {H}^d$, respectively. A geometric intuition of how the local intrinsic geometry changes in these models is given by the behaviour of (geodesic) triangles – see Fig. 5 for a visual representation in dimension 2 . We refer to^6^ for an advanced discussion on more detailed geometric properties.

Being positively curved, as opposed to being negatively curved, means that the (data) manifold “looks like” a sphere, as opposed to a hyperbolic space, and this comes along with particular (typically) local structural properties, and, ultimately, also functional consequences for the system at hand. Most real-world networks seem to have an underlying hyperbolic geometry, which is believed to be related to the fact that “networks that conform to hyperbolic geometry are maximally responsive to external and internal perturbations”^7^. Hyperbolic geometry, in fact, is thought to be related to faster information transmission^7,8^ and some real-world networks have already shown hyperbolic features; e.g. financial networks^9^, olfactory systems^10^, or brain-to-brain coordination networks^11^. Also functional brain networks were found to be best represented in hyperbolic spaces^12^.

Our analysis starts from these considerations, and aims to provide a purely qualitative topological reasoning justifying the observation that some complex systems have an intrinsic hyperbolic geometry. Investigations of data manifolds, from a Riemannian geometric perspective, need to *assume* a priori a certain geometry of the underlying data and, as in the case of the sphere, also a specific topology. This leads to the natural questions: what are the main differences in the topological features of data with intrinsic Euclidean and/or non-Euclidean underlying structure? And can we use this knowledge to (at least qualitatively) infer that certain real-world data have intrinsic non-Euclidean geometry, as it has been reported?

To investigate these questions, there are many possible approaches. In^3^, for example, methods from differential geometry were borrowed, and the authors aim to compute the curvature of data from the second fundamental form (after additional preprocessing and neighbourhood selection). In the current paper instead, we borrow tools from a recent subfield of algebraic topology, called Topological Data Analysis (TDA)^13^, hence, combining geometric and topological methods. The goal is achieved by employing manageable topological invariants such as homology and homotopy groups. The structure of the data is then qualitatively and quantitatively assessed in the form of topological features (e.g. connected components, voids, tunnels or loops). *Persistent Homology* (PH) is one of the main adopted tools, in TDA, to make assessments precise. PH is a multiscale adaptation of the classical homology theories, and it allows a computation of the *persistent* topological features of a space at all different resolutions, while revealing the most essential ones. It has provided novel qualitative and quantitative approaches to study complex data (in the form of either point clouds, time series or connectivity networks). Its direct use, along with its derived features, has already been beneficial in many fields, like neuroscience^14,15^, finance^16^, image classification ^17^, to name a few.

In^18^, a new PH-derived tool to *reliably detect signatures of structure and randomness that are invariant under non-linear monotone transformations* was introduced, called *Betti curves*. These features encode information of the topological and potentially also of the geometrical properties of data, represented by (weighted) graphs, as functions – curves – of the edge densities. Given an edge-weighted graph *G* representing the dataset of interest, we can apply thresholds to its edge weights. This yields a collection of undirected graphs $\{G_\alpha \}_{\alpha }$, where each graph corresponds to a different threshold level. For each $G_{\alpha }$, we then compute the ranks of certain homology groups: in dimension 0, these correspond to the number of connected components, and in dimension 1, these are related to the number of independent cycles. Collecting these values across all scales gives a high-dimensional feature vector: the *Betti curve* of *G*.

Our main interest in Betti curves comes from the fact that they distinguish geometric Euclidean networks from the random ones^18^. Furthermore, in dimension $\le 2$, persistent homology and Betti curves detect the curvature^19–21^ of the underlying manifold, and can be used for obtaining bounds on the Gromov-Hausdorff distance^22^. Inspired by these recent advancements, in this work we first explore and investigate the behaviour of Betti curves associated to random sample points uniformly distributed on the standard Riemannian manifolds (in spaces of arbitrary dimension) of sectional curvature $1,0,-1$, and we compare them with the Betti curves of random graphs. For Betti numbers in dimensions 0 and 1, as usually considered in applications, we show that the Betti curves do distinguish the three different geometries, hence the underlying manifolds. Aiming to use this gained qualitative information in understanding data manifolds, we proceed with analysing three main datasets, coming from brain, stocks, and climate data. These data usually come in the form of correlation matrices, hence we also analyse correlation networks. We then make use of the Betti-curves associated to the sphere, to the Eunclidean space, and to the hyperbolic space, as purely descriptive references. We observe a general hyperbologenic character, with brain and climate data derived features in between of those derived from the Euclidean and the hyperbolic geometry of small curvature; the topological features associated to the stocks data having an even more pronounced tendency towards hyperbolic behaviour. This observation is consistent with the general belief that these data, and in particular the stock market, have an intrinsic hyperbolic geometry. On the other hand, having similar Betti curves does not mean that the underlying geometries are equivalent, also in view of the high symmetry of the reference models used in the analysis. Further, the hyperbologenic effect could be caused by other factors not directly related to the curvature. Then, to gain more insight into how the observed results could have been affected by other factors, such as the modular structure – cf. Curto et al.^23^ – we include a study of the dependence of Betti curves on the number of modules. We observe a qualitative trend going from a Euclidean-like behaviour, in the case of a single module, to hyperbolic, when considering a few modules, to spherical.

To conclude, we believe that the methods investigated in this work can provide useful descriptors, complementing alternative approaches to analyse the connections between PH and the underlying curvature on manifolds^23,24^ and foresee that quantitative methods based on these approaches will gradually infiltrate data analytic practice similarly as other tools of TDA already have.

We now first give a description of the results and discussion, then in Section "Data description" we describe the datasets employed in this work, and then provide a brief account of the mathematical methods. For each dataset (in the form of time series), we apply the pipeline constructing the Betti curves as described in Section "On Betti curves of symmetric matrices" to their associated (Pearson) correlation graphs.

## Results

Given an edge-weighted graph *G*, we can use methods from topological data analysis to construct feature vectors amenable to machine learning algorithms. Although the main construction of Betti curves, studied in our experiments, is given in Section "Betti curves", we briefly recall here the main steps. First, assuming that *G* represents the distance matrix, we threshold *G* across the different weights of its edges – from min to max. This yields a a collection $\{G_\alpha \}_{\alpha }$ of undirected graphs – one for each different weight. Then, we compute for each such graph the associated Betti numbers – that is, in dimension 0 and 1, the number of connected components and of independent cycles of each $G_\alpha$. This way we obtain feature vectors of Betti numbers, one for each chosen dimension, that are called the *Betti curves* of *G*. In some experiments, we shall also use the *area under the curve* of the Betti curves, to be thought of as the integral of the curves.

The behaviour of the Betti curves associated to *random* graphs has been thoroughly investigated, and it is nowadays theoretically understood – see, e.g.^25,26^. A more geometric source of (symmetric, random) matrices is given by *geometric matrices*: instead of considering matrices with random entries, one first considers uniformly distributed random points lying on a geometric manifold (e.g. the hypercube $I^n$ or the sphere); then, one considers the distance matrix *A* obtained using the underlying geodesic distances between points $\{x_1,\dots ,x_N\}$, i.e. setting $A_{i,j}{:}{=}d(x_i,x_j)$. Observe that such distance matrix can be interpreted as a edge-weighted graph, hence we can compute the Betti curves. Betti curves associated to sample points in Euclidean spaces have been investigated by Giusti et al.^18^. They show that the Betti curves can distinguish random matrices from the geometric (Euclidean) ones.

### Geometric analysis

**Fig. 1:**
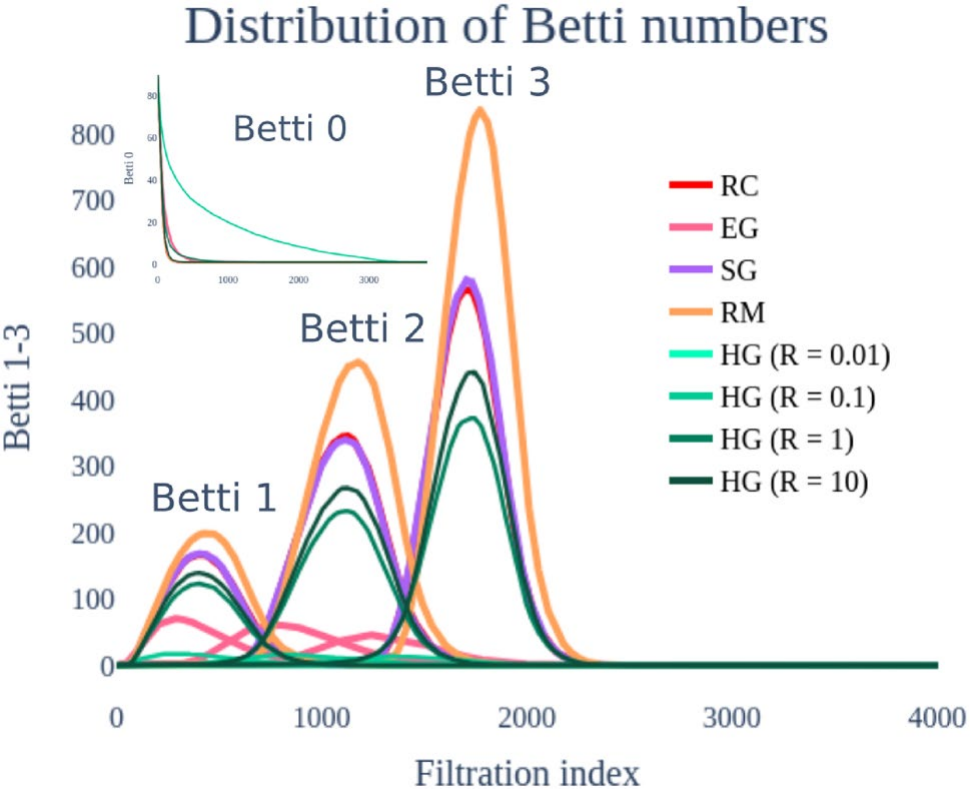
Comparison of Betti numbers distributions of simulated random matrices (RM), Euclidean geometry (EG), spherical geometry (SG), hyperbolic geometry (HG), and random correlation (RC) matrices. The sample size/dimensionality is 400. The Betti curves corresponding to the random correlation are almost not visible on the graph as it underlays the spherical geometry curve.

We start our analysis by extending the results of ^18^ to spherical geometry (i.e. points randomly distributed on the sphere $\mathbb {S}^n$, endowed with the spherical distance) or hyperbolic geometry (i.e. points randomly distributed on the hyperbolic space $\mathbb {H}^n$ with the hyperbolic distance). In order to randomly distribute points on such manifolds, we use uniform distributions; a uniform distribution of points in the hypercube $I^n$ in the Euclidean case, and a uniform distribution on the sphere in the spherical case. For the hyperbolic case, we use the Poincaré disc model, and the approximation of the distribution – see, e.g. ^27^ – at given radii. Similar choices were also investigated in^10^. In Fig. 1, we plot the obtained Betti curves in dimension 0, 1, 2, 3 associated to random distance matrices (Euclidean: EG, spherical: SG, hyperbolic at different radii: HG), random matrices RM and random correlation matrices RC (i.e. sample correlation matrices of Gaussian white noise samples).

#### Dependence on dimension

As apparent from Fig. 1, Betti curves distinguish not just random from Euclidean geometric matrices, but they are good descriptors for other geometries as well. Figure 1 represents in fact the average Betti curves (over 100 random iterations) in dimension 0, 1, 2, and 3, of $90\times 90$ random matrices, and geometric matrices of 90 sample points randomly distributed in the mentioned geometric manifolds of dimension 400. In the case of the hyperbolic space, we considered radii $R=0.01, 0.1,1,10$ – see ^27^ and the discussion below about the role of *R*. We complemented it with the Betti curves of random correlation matrices with 90 time series of length 400 – the typical length of fMRI data. Under these choices, the higher-dimensional ($\ge 1$) Betti curves associated to random matrices show the highest peaks, followed by the Betti curves of points randomly distributed on the sphere first, in the Euclidean space then, and in the hyperbolic space last.

Note that the described qualitative behaviour holds across a range of dimensions of the manifolds (which corresponds to the time series length or sample size) and the number of points sampled, although for small dimensions the order can be reversed. In^18^, it was observed that correlation matrices of finite samples of *N* independent uniformly distributed random variables display the same characteristic Betti curves as random symmetric $N\times N$ matrices (note that for correlating spike trains, the mean of a cross-correlogram was used by^18^). However, in our results (for time series of length 400) illustrated in Fig. 1, the characteristics of random correlation (RC) matrices do not correspond to the random matrices (RM). We suggest that the observation presented in^18^ holds only for sufficiently high dimensions (they used 10000 samples), an observation which might be relevant for the interpretation of TDA analysis of small datasets common in many disciplines.

**Fig. 2:**
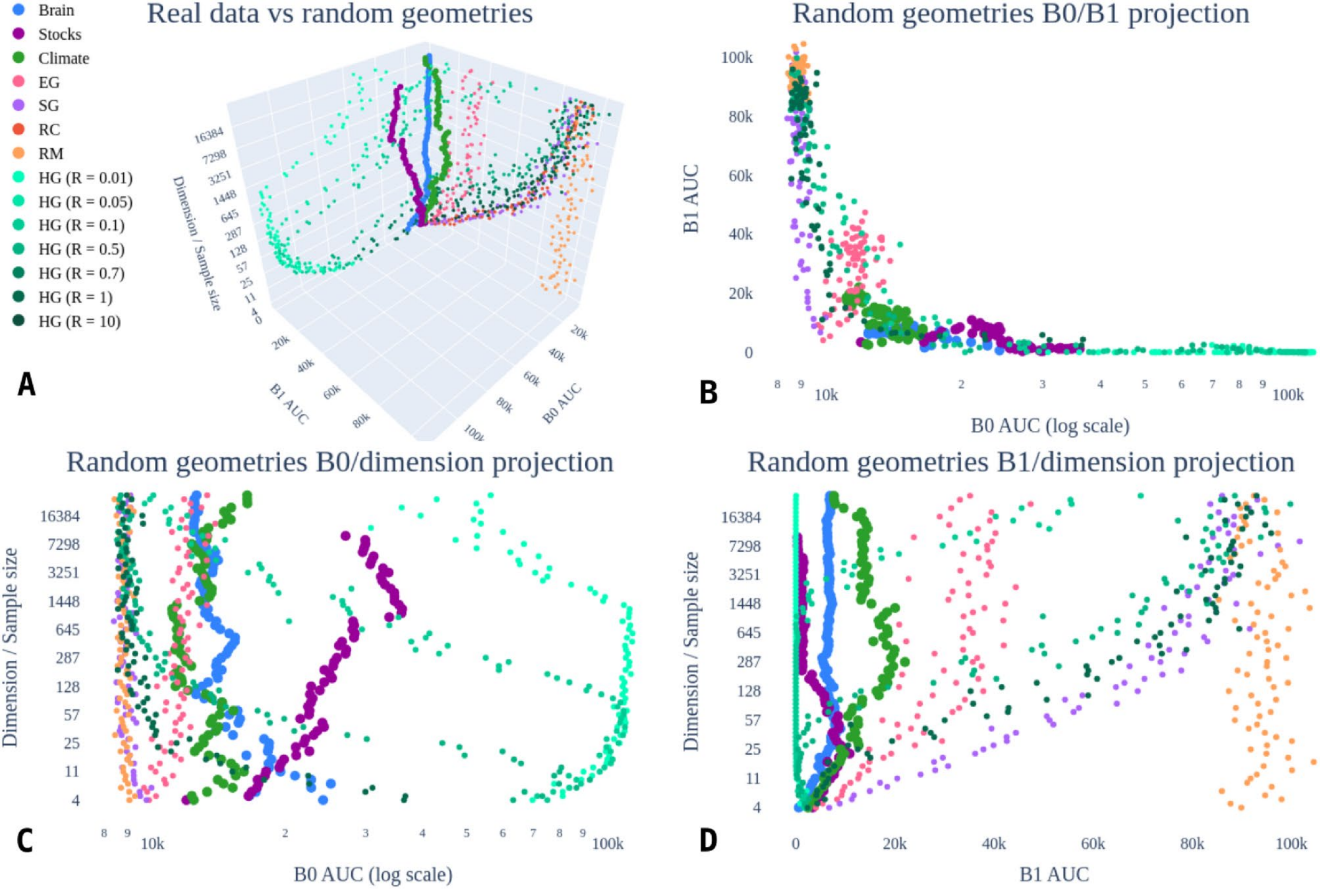
Integral Betti signatures for real data correlation matrices, random matrices (RM)/Euclidean geometry (EG)/spherical geometry (SG)/hyperbolic geometry (HG)/random correlation (RC) matrices. Subfigure **A** shows the 3D plot of integral Betti signatures for different sample sizes/dimensionality for the real data and the simulated random geometries. Subfigures **B**, **C** and **D** present the 2D projections B0AUC/B1AUC, B0AUC/dimensionality and B1AUC/dimensionality, respectively.

In Fig. 2, we show the 3-dimensional plot in the coordinates (B0 AUC, B1 AUC, sample size) corresponding to the integral Betti signatures (the Area Under the Curve of the 0-th and 1-st Betti curves), along with the dimension of the manifold (sample size). We plot the features corresponding to the three datasets described in Section "Data description", and to the random matrices, geometric (Euclidean, spherical, hyperbolic) matrices, and correlation matrices. All datasets have TS length up to $2^{15}$, giving an overview of the asymptotic behaviour of the associated Betti curves. The random/geometric curves are very distinct from each other; correlation is close to them for short samples, but converges to random for longs samples.

#### Random correlation and spherical geometry

We observe that the Betti curves of spherical and correlation matrices are similar. This is because correlation matrices have an intrinsic spherical geometry. Indeed, the Pearson correlation of *n*-dimensional random variables yields the normalized scalar product between the corresponding vectors in $\mathbb {R}^{n-1}$; from which one obtains the angle between such vectors by application of the function $\textrm{arccos}$. Hence, it is equivalent to the spherical distance between them, explaining the similar behaviour of their Betti curves shown in Figs. 1 and 2.

#### Hyperbolic geometry

We complete our analysis of Betti curves of geometric matrices by investigating also the behaviour of point clouds distributed in the hyperbolic space $\mathbb {H}^n$. The choice of radii was $R=0.01,0.05,0.1,0.5,0.7,1,10$. On the technical note, the random points in the hyperbolic space $\mathbb {H}^n$ have been generated in the Poincaré disc model. For a given $R\in (0,\infty )$ we have randomly generated vectors in the ball $B^n_R$ of vectors of norm $\le R$. The obtained points belong to the hyperbolic space of radius *R*; we have then projected such points on the standard hyperbolic space $\mathbb {H}^n$ by applying the transformation $r\mapsto (\cosh {\rho (r)} - 1)/(2 + \cosh {\rho (r)})$; see also the Supplementary Information.

For such configurations, for small radii we observe that the Betti numbers $\beta _p$, for $p>0$, are close to 0 (see Fig. 1). When the radius increases, also B0AUC and B1AUC increase, showing characteristics closer to random correlation. This effect holds consistently across the choice of dimension, albeit more visible for higher dimensions (see Fig. 2D). Note also from Fig. 1 that different radii have different behaviours of 0-th Betti curves, with lower slope for smaller radii.

Overall, the behavior of the Betti curves depends on the choice of the radius *R*. For sufficiently small *R*, the number of cycles (that is, the values of $\beta _1$) remains close to zero. In what follows, we will refer to this regime, where almost no cycles are present, as exhibiting *hyperbolic character*.

#### Random geometries separability

**Fig. 3:**
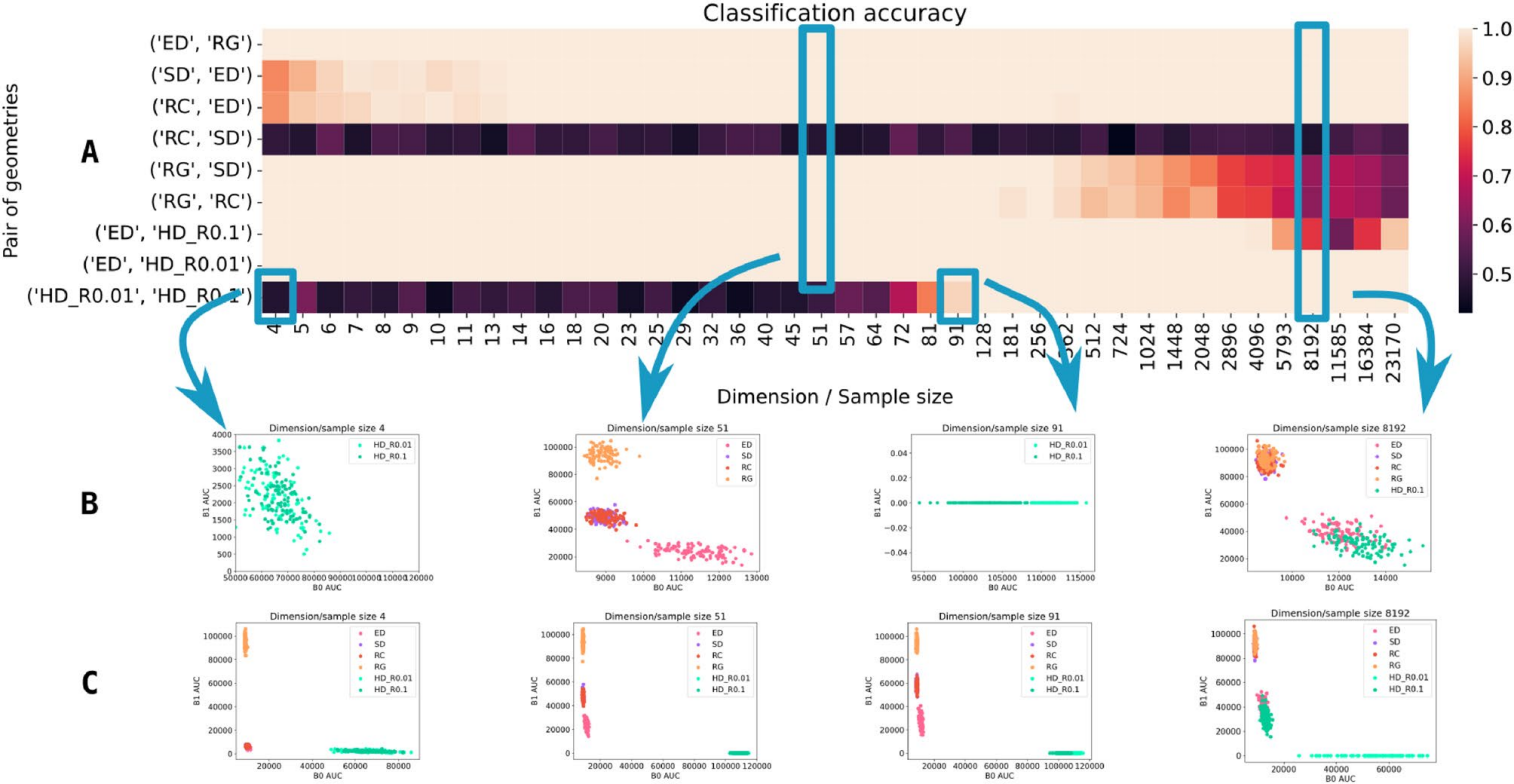
Separability of random geometries across different dimensions. (**A**) Classification accuracy between pairs of geometries. Some pairs are consistently well separated across all dimensions (e.g., Euclidean vs. Random Graph), others are not separable (e.g., Random Correlation vs. Spherical), while some are separable only in selected dimensions. (**B**) Zoomed-in scatter plots of different geometries for selected dimensions. (**C**) Same scatter plots as in (**B**), but shown with the full scale.

To characterize the random geometries’ separability quantitatively, we used more extensive simulations. For every geometry and every dimension, we generate 100 points and computed B0AUC and B1AUC. Then, we pairwise run the linear Support Vector Machine classifier to distinguish the geometries ($50/50\%$ train-test split). The obtained accuracy is presented in Fig. 3. As expected from the plots in Fig. 2, some pairs, such as Euclidean and random, are consistently distinguishable, while others, such as random correlation and spherical, remain indistinguishable for the classification. Notably, certain geometries are (in)distinguishable only in specific dimensions, reflecting natural limitations of the low-dimensional B0AUC/B1AUC representation.

### Curvature of real-world data

We analyse the case of three main sources of different nature: brain, stocks, and climate data; these are described in Section "Data description". In order to analyse the influence of the sample size on the associated Betti curves, for every dataset, we start with an initial segment of length 4 (extremely short and not meaningful in practice, but illustrative concerning the general effects), and then take longer segments until $2^{15}$ points (very long, longer than used in many practical applications). We also consider (simulated) random and geometric matrices as comparison models; the size of these matrices is also fixed to $90\times 90$, with sample points taken from manifolds of dimensions corresponding to the sample size. In Fig. 2 we show the associated 3-dimensional plot. We wish to point out here that there is no theoretical reason why the underlying geometry of the considered data should be related to highly symmetric geometries such as the Euclidean/symmetric/hyperbolic ones. On the other hand, such geometries are the few ones amenable to computations, which is the main reason why we chose them for the described comparative analysis of the Betti curves.

As discussed in the previous subsections, the Betti curves of random matrices are quite distinct from those of any of the geometric matrices, at least for low dimensions (sample size). For small radii, the hyperbolic matrices are close to 0 in the B1AUC-coordinate – related to the fact that for small radii, there are few 1-dimensional cycles. On the other hand, for large radii the Betti curves of the hyperbolic geometric matrices are comparable with those of random correlation and spherical geometric matrices – particularly so in high dimensions.

For random matrices, the point cloud is approximately normally distributed in the B0AUC-B1AUC projection, and there is no substantial dependence on the dimensionality. In contrast, the Euclidean geometry shows a less pronounced but still present systematic effect of sample size.

Turning to real data, we observe that its topological features sit clearly between hyperbolic (of small radii) and Euclidean features. The longer the time series, the more stable the Integral Betti signatures become, and has a smaller value of B1AUC (fewer 1-dimensional cycles). Particularly, the stock data show few 1-dimensional cycles represented in the B1AUC coordinates, and it seems to be closer to the hyperbolic distributions (with small radii). Also, the behaviour in B0AUC changes with the sample size. Note that the behaviour of the stock data shown in Fig. 2 agrees with previous discussions on the hyperbolic representation of the stock market^28–30^ and that this is also consistent with part of the results of^31^. We point out that both the stock market^32^ and climate^33^ time series contain non-negligible nonstationarities, while fMRI data are concatenated across subjects; this may contribute to some visible jumps in the integral Betti signatures when changing time series length. This gets smoothed out when a stationary model of the stock data is sampled (simulations not shown), making even clearer the general drift with increasing sample size towards an apparent low-radius hyperbolic geometry.

Despite the similarity of the analysed Betti curves to hyperbolic geometry, particularly in the sense of absence of cycles, the data do not strictly line up with either of these geometries (see Figure S.1 in the Supplementary information for more details). Although being possibly related to an intrinsic hyperbolicity of the system, our results might also be related to other characteristics of the datasets; e.g. to a small rank property, whose effect on the Betti curves was theoretically investigated in^23^. In the last part of our experiments, we focus on this effect.

### Modularity

**Fig. 4:**
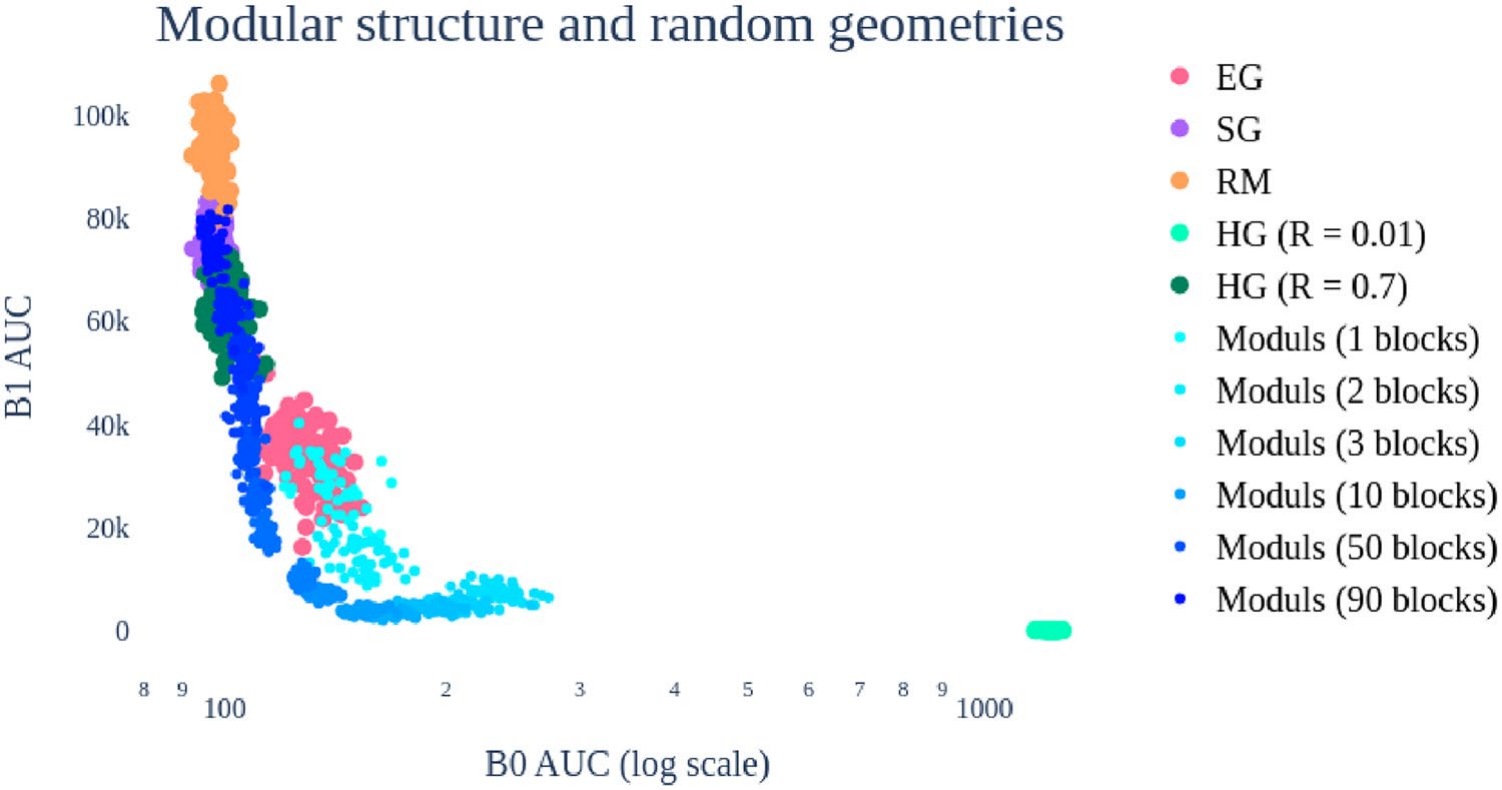
Integral Betti signatures of geometric distributions and modular systems (for a growing number of modules). Shortcuts: EG - Euclidean geometry, SG - spherical geometry, RM - random matrices, HG - hyperbolic geometry. The sample size/dimensionality is 400.

We aim to understand the dependence of the Betti curves with respect to (small) rank matrices. In order to create (small) rank matrices we have constructed modular systems with modules of the same size. To do so, we start with 90 independent random time series (white noise) with the length of 400 sample points; for constructing a modular system of size $m\le 90$, we first select *m* of such time series; say $M_1,\dots ,M_m$. We consider the vector $[M_1,\dots ,M_m,M_1,\dots ,M_m,\dots ,M_1,\dots ,M_{k}]$ where $k \equiv 90 \mod m$, and in total we create 90 time series with *m* modules of approximately the same size. After copying the time series, we add a small-amplitude white noise. The effect of this modular structure – projected onto the B0AUC-B1AUC plane – is shown in Fig. 4; here, we plot features for the random and geometric distributions, along with those associated to the described matrices with *m* modules, for $m=1,\dots , 90$.

The modules show a gradient starting close to Euclidean (corresponding to 1 module) and then moving to hyperbolic (until about 5 modules). The gradient stays in the “hyperbolic regime” (at different radii) and then continues towards random (when reaching the full rank).

This simulation demonstrates that, depending on the number of modules, the Betti curves in the (B0AUC, B1AUC) coordinates yield features which are similar to certain specific geometric matrices; going from Euclidean to hyperbolic of different radii to spherical to random. When the modular system is described by a single module, this behaviour is explained by the fact that, locally, the spherical geometry looks like Euclidean in one less dimension (consider points sampled close to a pole of a sphere effectively close to Gaussian sample from the tangential plane). When the number of modules increases (but still far from yielding a full rank matrix), the shift to the hyperbolic direction of small radii (hence, of few 1-cycles) can be explained by the features being “governed” by the behaviour of only a small amount of points on the sphere, the centroids provided by the modules themselves; there is a shift to bigger B0AUC, determined by the fact that, overall, the number of sample points is still higher than the number of centroids. When the number of modules goes to the full matrix rank, the distribution on the sphere tends to be more homogeneous, hence behaving like the random distribution. This effect depends on the dimension of the space.

## Discussion

Betti curves are well understood from a theoretical point of view, and were shown to provide an effective tool in distinguishing random from (Euclidean) geometric matrices ^18^. The asymptotic behaviour of the Betti curves associated to long time series was also investigated^18^. However, in practical applications, one commonly deals with short time series. We showed that the qualitative behaviour of Betti curves may change considerably. We also extended the analysis previously developed in^18^ (in the case of random and Euclidean geometry) to spherical and hyperbolic geometries. We have used the AUC in conjunction with the 0 and 1-dimensional Betti curves as main qualitative measures, which we referred to as *integral Betti signatures*. The outputs depend on several parameters; first, the number of points sampled and the dimension of the ambient manifolds. We investigated the dependence on these parameters, showing that the Betti curves distinguish the three geometric models. We have considered the three geometries – Euclidean/spherical/hyperbolic – as base comparison models in the subsequent analysis of real data. The choice of these models is driven by their amenability to computations – and in particular of the Betti curves of random points distributed in such models. We would like to emphasize that there is no reason for the data to be related to any of those highly symmetric geometries, and the analysis should be regarded as purely comparative.

In such comparative experiments, applying Pearson correlation to obtain symmetric matrices, we observed that Betti curves associated to real world time series sit in between hyperbolic far from being spherical. On the other hand, for larger radii, hyperbolic geometric matrices seem to behave like spherical ones. This is intriguing, given that truly random correlation matrices behave rather like spherical geometric matrices. Data indeed can have true hyperbolic underlying geometry, as shown across contexts^9–11^, but this “hyperbologenic effect” can also be due to other factors; for example, it can be an artefact due to small data rank. Following the theoretical study in^23^, we analysed the small rank effect on Betti curves in simulations, showing how the qualitative trends depend on the number of modules. As suggested to us by a referee, the “hyperbologenic effect” could also depend upon the exponential volume growth, and this is open to be investigated. Moreover, we observed the role of the choice of the metric adopted in the construction of the order complexes. In Supplementary Figure S.2, we show how the Betti curves of the random geometric matrices change when using a different metric (defined on the same underlying manifold). Also, the ordering of the filtration in the construction of the order complexes matters. This shows again how the metric (rather than solely the distribution of sample points) plays an important role in the computation of Betti curves. For real data (see Supplementary Figure S.3), the dependence on the distribution of points is deeper – e.g. for climate and brain. The main differences are visible for *shorter* time series, for lengths typical for real-world scenarios. Finally, we study the Betti numbers given by the Pearson correlation as a measure of dependence, a choice shown to capture sufficiently pairwise dependence in brain ^34^, climate^33^ and financial^32^ data. While the correlation matrix captures rich (multivariate) global structure, high-order interactions are of interest^35^, although e.g. in the case of brain not dominant^36^.

We now turn to our main motivation. When dealing with complex networks, it is believed that most complex networks have some hidden network geometry^37^. There is a general belief that further investigations of such hidden geometries will deepen our understanding of the fundamental laws describing relationships between structure and function of complex networks^38^. However, connections between the hidden geometry and the combinatorial properties of the networks are not yet totally understood. Some works have partially described these relations, e.g.^37^ in the context of emergent hyperbolic geometry in growing simplicial complexes. However, a complete picture (for real data) is still missing. Persistent homology and Betti curves have proved to be great qualitative tools in investigating related questions. In fact, geometric properties, like the curvature, have effects on the homology of the underlying manifolds^39^, hence, on persistent homology. Moreover, PH-derived features are easily computable, thanks to various algorithms freely available. However, more relations between persistent homology and the curvature of data manifolds have yet to be discovered; see the recent advances^19–23^. Our analysis is meant to contribute to the advancing of our understanding of the hidden structure of data manifolds; we believe that PH- and TDA-based approaches will be beneficial for these tasks, as we qualitatively showed in our results.

## Materials and methods

### Data description

#### Brain data

We analyse the dataset that consists of fMRI recordings of 90 healthy controls. Functional MRI data were collected with a 3T MR scanner (Siemens; Magnetom Trio) at Institute of Clinical and Experimental Medicine in Prague ($T2^*$-weighted images with BOLD contrast, voxel size 3$\times$3$\times$3mm^3^, TR/TE = 2000/30ms, 400 time points). Initial data preprocessing was done using FSL routines (FMRIB Software Library v5.0, Analysis Group, FMRIB, Oxford, UK) and CONN toolbox (McGovern Institute for Brain Research, MIT, USA). See ^40^ for detailed prepocessing descriptions; the data itself can be found in the online repository ^41^ https://data.mendeley.com/datasets/crx7d22pym/4. To extract the time series for further analysis, the brain’s spatial domain was divided into 90 non-overlapping regions of interest (ROIs) according to the Automatic Anatomical Labelling (AAL) atlas^42^; from each ROI we extract one BOLD time series by averaging the time series of all voxels in the ROI.

#### Climate data

We use the daily surface air temperature anomalies data obtained from the NECP/NCAR reanalysis dataset^43^ (https://psl.noaa.gov/data/gridded/index.html). In particular, we use the daily air temperature fields at 1000hPa level, spanning the period from 1/1/1948 to 12/31/2012 and sampled at $2.5^\circ \times 2.5^\circ$ angularly regular Gaussian grid. For a more precise description of the dataset, we refer to^44^. The resulting time series has a length of 23376 time points at each of 162 geodesic grid nodes. We randomly select 90 out of the 162 time series for better comparability with the brain data.

#### Stocks data

We use historical stock prices, downloaded from the Yahoo! Finance service^45^, belonging to the New York Stock Exchange 100 (NYSE100) index. We consider only stocks traded between 4 January 1977 and 6 October 2023. This restriction results in $N = 90$ stocks. For daily data, this leads to a data length of $T = 11791$. We used the daily adjusted closing prices. The *logarithmic return* is computed as the first differences of the log-transformed prices: $r_i(t) = \log \left[ \frac{p_i(t)}{p_i(t-1)}\right] ,$ where $p_i(t)$ is the (adjusted, daily) closing price of stock *i* at time *t*. For a more detailed analysis of a similar dataset, see ^32^.

### On Betti curves of symmetric matrices

The topological pipeline employed in this work was first introduced by Giusti *et al.* in^18^. For a given symmetric (real-valued) matrix, we consider certain topological descriptors called *Betti curves* – cf. Figure 1. In order to concisely represent Betti curves, and for comparisons, we use their associated *Area Under the Curve* (AUC), and call the corresponding features *integral Betti signatures.*

#### Betti curves

Betti curves are topological descriptors of symmetric matrices. To each $N\times N$ symmetric matrix *M* with distinct non-zero real-valued entries, we first associate a sequence of graphs

1 $$\begin{aligned} \textrm{ord}(M){:}{=}G_0\subseteq G_1\subseteq \dots \subseteq G_k \ , \end{aligned}$$

called the *order complex* $\textrm{ord}(M)$ of *M*^18^, Def. 2, SI. The construction of $\textrm{ord}(M)$ starts with a totally disconnected graph $G_0$ on *N* vertices, and proceeds step by step by adding new edges; addition of edges follows the values of the entries of *M*, sorted either min to max or max to min. We refer to the supplementary notes for a more precise description of the order complex; note that in all the applications below, values are sorted from the minimum to the maximum value while assuming the distance matrix as input. To generate the distance matrix from the correlation, we apply $1-M$ for correlation matrices before computing the filtration. In this way, we obtain a sequence of (undirected) graphs, as in Eq. 1. To each graph $G_i$ in $\textrm{ord}(M)$, we associate a simplicial complex $\widetilde{G_i}$, called the *clique complex* of $G_i$, whose *p*-simplices consist of all the complete subgraphs on $(p+1)$ vertices of $G_i$ – see Definition A.5. in the Supplementary information. This yields the sequence of clique complexes

2 $$\begin{aligned} \widetilde{G_0}\subseteq \widetilde{G_1}\subseteq \dots \subseteq \widetilde{G_k} \ , \end{aligned}$$

associated to *M*. Classical topological invariants of simplicial complexes are the *homology groups* – see, e.g. ^46^ and the supplementary – along with their ranks, called *Betti numbers*. The number of *p*-holes of a simplicial complex $\Sigma$ is related to the *p*-th Betti number $\beta _p(\Sigma )$ associated to $\Sigma$. Specifically, the 0-th Betti number $\beta _0$ of a simplicial complex gives the number of connected components, and the 1-st Betti number $\beta _1$ the number of independent loops.

For an order complex $\textrm{ord}(M)$, and for each $i\in \mathbb {N}$, we can compute the *i*-th Betti number of the clique complexes $\widetilde{G_j}$ appearing in Eq. (2). Hence, we obtain a sequence of natural numbers

3 $$\begin{aligned} \beta _i(\widetilde{G_0}), \beta _i(\widetilde{G_1}), \dots , \beta _i(\widetilde{G_k}) \end{aligned}$$

consisting of the *i*-th Betti numbers $\beta _i(\widetilde{G_j})$, and called the *i*-th *Betti curve* of *M*. For instance, when $i=0$, the Betti number $\beta _0(\widetilde{G_j})$ computes the number of connected components of $G_j$. Therefore, the 0-th Betti curve of *M* is given by the vector of connected components of the graphs $G_j$, with *j* varying across the filtration parameter. Analogously for $i=1$, where the 1-st Betti curve is related to the evolution of the cycles of the graphs $G_j$, or for the higher *i*-th Betti curves.

#### Random, geometric and correlation matrices

We call *random* any symmetric matrix with identically independent real-valued entries, *geometric* any symmetric matrix which is obtained as the distance matrix of sample points randomly distributed on a manifold, and *correlation matrix* any symmetric matrix obtained as the (Pearson) correlation matrix of given time series. When dealing with geometric and correlation matrices, we shall consider the case of points sampled from the fundamental metric spaces $\mathbb {R}^n$ (Euclidean space), $\mathbb {S}^n$ (a sphere), and $\mathbb {H}^n$ (a hyperbolic space). It is known that these are the geometric models of the simply connected *n*-manifolds of constant sectional curvature 0, 1 and $-1$, respectively.

**Fig. 5:**
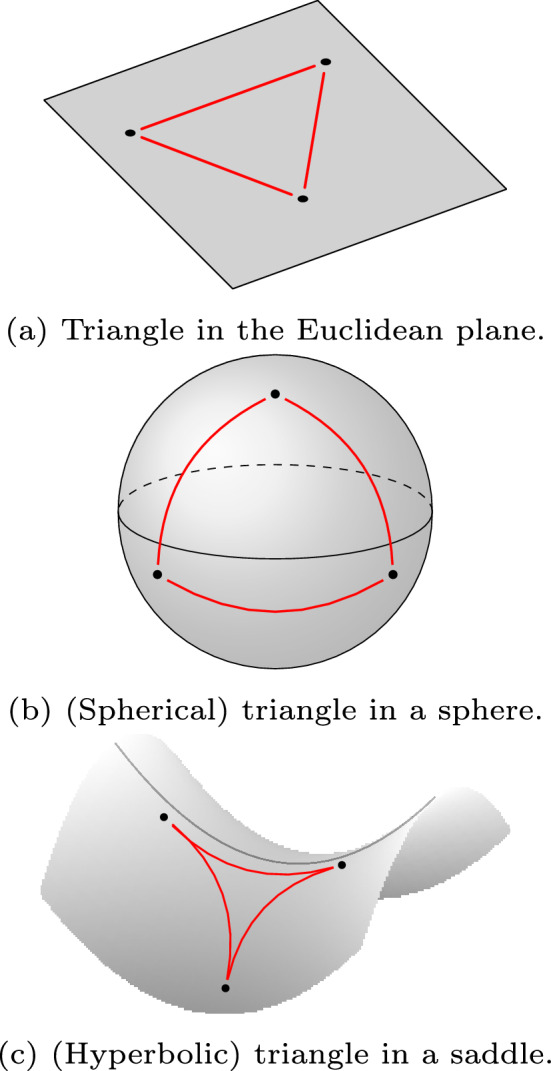
Standard geodesic triangles in 2-dimensional spaces of zero, positive or negative (sectional) curvature.

Generally speaking, the geometric properties of a manifold, endowed with different underlying metrics, can be very different. First and foremost, geodesic triangles in the three fundamental models, have different behaviours: if triangles in the Euclidean space are “straight”, they get “fatter” on a sphere and “thinner” in a space of negative curvature – see Fig. 5 for a visual representation in dimension 2 . An analytical description of the metrics in these geometries is given in the Supplementary. In practical examples, and in our results, we consider sample points randomly distributed in the unit cube, on the unit sphere or in the hyperbolic space – see the Supplementary, and^47^ for possible hyperbolic models – and then compute the Betti curves described in Section "Betti curves".

#### Dimensional reduction by AUC

In order to compare the Betti curves of different datasets, we consider two intrinsic geometric measures: the Area Under the Curve (AUC) of the Betti curves $\beta _0$ and $\beta _1$. More specifically, the AUC of the *i*-th Betti curve is given by its integral – that is the AUC is the sum of the Betti numbers $\beta _i$. In fact, as shown in Fig. 1, the qualitative behaviours of the Betti numbers in dimension $i\ge 1$ are all similar, whereas in dimension 0 they are not. The AUC has been chosen for taking into account the trend of the number of *k*-cycles in a given dimension *k*, up to affine transformations (as the AUC is invariant under shifts). We will call them *integral Betti signatures*. Therefore, for each dataset, at different sample sizes, we compute the integral Betti signatures, obtaining 3-dimensional representations – see Fig. 2.

## Conclusions

As highlighted earlier, high-dimensional datasets are important in many scientific domains, including biology, economics or climate. Data are increasingly assumed to lie on hidden geometric manifolds. Therefore, understanding geometric and topological properties, such as the curvature, of these manifolds can be beneficial for uncovering hidden structures of data. Estimations of the curvature have been previously considered with analytical methods. In this work, we further developed the integral Betti signature approach based on modern tools from Algebraic Topology and Topological Data Analysis, documenting its ability to characterize the differences, as well as interesting similarities, among topologies of a range of random and geometric distributions. Importantly, we showed that these relations depend crucially and systematically on further aspects including sample size/space dimension, hyperbolic space radius, and artificially imposed modularity; features that need thus to be taken into account when interpreting observed Betti signatures. Finally, application to real-world data demonstrated that the Betti curves of brain, climate, and financial datasets tend to be the closest to the Betti curves of hyperbolic, i.e. fast-transmission, networks. This does not show that the data are indeed hyperbolic, as such characteristics can depend on other factors such as modularity.

## Supplementary Information

Supplementary Information.

## Data Availability

The datasets used and/or analysed during the current study available from the corresponding author on reasonable request.

## References

[1] Fefferman, C., Mitter, S. & Narayanan, H. Testing the manifold hypothesis. *J. Amer. Math. Soc.* **29**(4), 983–1049 (2016).

[2] Carlsson, G., Ishkhanov, T., de Silva, V. & Zomorodian, A. On the local behavior of spaces of natural images. *Int. J. Comput. Vis.* **76**(1), 1–12 (2008).

[3] Sritharan, D., Wang, S. & Hormoz, S. Computing the Riemannian curvature of image patch and single-cell RNA sequencing data manifolds using extrinsic differential geometry. *Proceedings of the National Academy of Sciences* **118**(29), e2100473118 (2021). 10.1073/pnas.2100473118 PMC8307776 34272279

[4] Lin, B., He, X. & Ye, J. A geometric viewpoint of manifold learning, * Appl. Inform.*, vol. 2, no. 3, (2015).

[5] Lin, T. & Zha, H. Riemannian manifold learning, *IEEE Trans. Pattern Anal. Mach. Intell.*, vol. 30, no. 5, (2008). 10.1109/TPAMI.2007.70735 18369250

[6] Bridson, M. R. & Haefliger, A. *Metric spaces of non-positive curvature* Vol. 319 (Springer-Verlag, Berlin, 1999).

[7] Sharpee, T. O. An argument for hyperbolic geometry in neural circuits. *Current Opinion in Neurobiology***58**, 101–104 (2019).31476550 10.1016/j.conb.2019.07.008

[8] Krioukov, D., Papadopoulos, F., Kitsak, M., Vahdat, A. & Boguñá, M. Hyperbolic geometry of complex networks. *Phys. Rev. E* **82**, 036106 (2010). 10.1103/PhysRevE.82.036106 21230138

[9] Keller-Ressel, M. & Nargang, S. The Hyperbolic Geometry of Financial Networks. *Sci Rep* **11**, 4732 (2021). 33637827 10.1038/s41598-021-83328-4 PMC7910495

[10] Zhou, Y., Smith, B.H. & Sharpee, T.O. Hyperbolic geometry of the olfactory space, *Science Advances*, vol. 4, no. 8, (2018). 10.1126/sciadv.aaq1458 PMC6114987 30167457

[11] Tadic, B., Andjelkovic, M. & Šuvakov, M. Origin of hyperbolicity in brain-to-brain coordination networks, *Frontiers in Physics*, vol. 6, (2018).

[12] Whi, W., Ha, S., Kang, H. & Lee, D. S. Hyperbolic disc embedding of functional human brain connectomes using resting state fMRI. *Netw Neurosci* **6**, 745–764 (2022). 36607197 10.1162/netn_a_00243 PMC9810369

[13] Wasserman, L. Topological data analysis. *Annual Review of Statistics and Its Application* **5**(1), 501–532 (2018).

[14] Lee, H., Chung, M., Kang, H., Kim, B.-N. & Lee, D. Discriminative persistent homology of brain networks, *Proceedings - International Symposium on Biomedical Imaging*, pp. 841–844, 03 (2011).

[15] Caputi, L., Pidnebesna, A. & Hlinka, J. Promises and pitfalls of topological data analysis for brain connectivity analysis. *NeuroImage* **238**, 118245 (2021). 34111515 10.1016/j.neuroimage.2021.118245

[16] Gidea, M. Topology data analysis of critical transitions in financial networks, *SSRN Electronic Journal*, 01 (2017).

[17] Dey, T. K. , Mandal, S. & Varcho, W. Improved Image Classification using Topological Persistence, in *Vision, Modeling and Visualization* (M. Hullin, R. Klein, T. Schultz, and A. Yao, eds.), (2017).

[18] Giusti, C., Pastalkova, E., Curto, C. & Itskov, V. Clique topology reveals intrinsic geometric structure in neural correlations. *Proceedings of the National Academy of Sciences* **112**(44), 13455–13460 (2015). 10.1073/pnas.1506407112 PMC4640785 26487684

[19] Bubenik, P., Hull, M., Patel, D. & Whittle, B. Persistent homology detects curvature. *Inverse Problems* **36**(2), 025008 (2020).

[20] Turkes, R., Montufar, G. F. & Otter, N. On the effectiveness of persistent homology, in *Advances in Neural Information Processing Systems* (S. Koyejo, S. Mohamed, A. Agarwal, D. Belgrave, K. Cho, and A. Oh, eds.), vol. 35, pp. 35432–35448, Curran Associates, Inc., (2022).

[21] Hacquard, O. & Lebovici, V. Euler characteristic tools for topological data analysis, (2023).

[22] Lim, S., Memoli, F. & Okutan, O. B. Vietoris-Rips Persistent Homology, Injective Metric Spaces, and The Filling Radius, (2020).

[23] Curto, C., Paik, J. & Rivin, I. Betti curves of rank one symmetric matrices, in *Geometric Science of Information* (F. Nielsen and F. Barbaresco, eds.), pp. 645–655, (2021).

[24] Fernández, X., Borghini, E., Mindlin, G. & Groisman, P. Intrinsic persistent homology via density-based metric learning. *Journal of Machine Learning Research* **24**(75), 1–42 (2023).

[25] Kahle, M. Topology of random clique complexes. *Discrete Mathematics* **309**(6), 1658–1671 (2009).

[26] Kahle, M. & Meckes, E. Limit theorems for betti numbers of random simplicial complexes. *Homology, Homotopy and Applications* **15**(1), 343–374 (2013).

[27] Alanis-Lobato, G. & Andrade, M. Distance distribution between complex network nodes in hyperbolic space, *Complex Syst.*, vol. 25, (2016).

[28] Sawhney, R., Agarwal, S., Wadhwa, A. & Shah, R. Exploring the scale-free nature of stock markets: Hyperbolic graph learning for algorithmic trading, in *Proceedings of the Web Conference 2021*, WWW ’21, (New York, NY, USA), p. 11–22, Association for Computing Machinery, (2021).

[29] Agarwal, S., Sawhney, R., Thakkar, M., Nakov, P., Han, J. & Derr, T. Think: Temporal hypergraph hyperbolic network, in *2022 IEEE International Conference on Data Mining (ICDM)*, pp. 849–854, (2022).

[30] Pawanesh, P., Sharma, C. & Sahni, N. Exploiting the geometry of heterogeneous networks: A case study of the indian stock market, (2024).

[31] Guidolin, A., Desroches, M., Victor, J. D., Purpura, K. P. & R. S. Geometry of spiking patterns in early visual cortex: a topological data analytic approach, *J. R. Soc. Interface*, vol. 19, no. 196, p. 20220677, (2022). 10.1098/rsif.2022.0677 PMC9667368 36382589

[32] Hartman, D. & Hlinka, J. Nonlinearity in stock networks. *Chaos: An Interdisciplinary Journal of Nonlinear Science* **28**(8), 083127 (2018). 10.1063/1.5023309 30180637

[33] Hlinka, J., Hartman, D., M. et al. Vejmelka, Non-linear dependence and teleconnections in climate data: sources, relevance, nonstationarity, * Climate Dynamics*, vol. 42, no. 7-8, pp. 1873–1886, (2014).

[34] Hlinka, J., Palus, M., Vejmelka, M., Mantini, D. & Corbetta, M. Functional connectivity in resting-state fmri: Is linear correlation sufficient?. *NeuroImage* **54**, 2218–25 (2011). 20800096 10.1016/j.neuroimage.2010.08.042 PMC4139498

[35] Rosas, F. E. et al. Disentangling high-order mechanisms and high-order behaviours in complex systems. *Nature Physics* **18**, 476–477 (2022).

[36] Martin, E., Hlinka, J., Meinke, A., Děchtěrenko, F., Tintěra, J., Oliver, I. & Davidsen, J. Network Inference and Maximum Entropy Estimation on Information Diagrams, *Scientific Reports*, vol. 7, no. 1, (2017). 10.1038/s41598-017-06208-w PMC5539257 28765522

[37] Bianconi, G. & Rahmede, C. Emergent hyperbolic network geometry, *Sci. Rep.*, vol. 7, (2017). 10.1038/srep41974 PMC5294422 28167818

[38] Boguñá, M., Krioukov, D. & Claffy, K. Navigability of complex networks, *Nature Phys*, vol. 5, (2009).

[39] Goldberg, S. I. *Curvature and homology*. Courier Corporation, (1998).

[40] Kopal, J., Pidnebesna, A., Tomeček, D., Tintěra, J. & Hlinka, J. Typicality of Functional Connectivity robustly captures motion artifacts in rs-fMRI across datasets, atlases and preprocessing pipelines. *Human Brain Mapping* **41**(18), 5325–5340 (2020). 32881215 10.1002/hbm.25195 PMC7670643

[41] Kopal, J., Hlinka, J. & Pidnebesna, A. Typicality of Functional Connectivity robustly captures motion artifacts in rs-fMRI across datasets, atlases, and preprocessing pipelines, *Mendeley Data*, vol. 4, (2024). 10.1002/hbm.25195 PMC7670643 32881215

[42] Tzourio-Mazoyer, N. et al. Automated Anatomical Labeling of Activations in SPM Using a Macroscopic Anatomical Parcellation of the MNI MRI Single-Subject Brain. *NeuroImage* **15**(1), 273–289 (2002). 11771995 10.1006/nimg.2001.0978

[43] Kalnay, E. et al. The NCEP/NCAR 40-year reanalysis project. *Bulletin of the American Meteorological Society* **77**(3), 437–471 (1996).

[44] Hlinka, J. et al. Reliability of inference of directed climate networks using conditional mutual information. *Entropy* **15**(6), 2023–2045 (2013).

[45] Yahoo! Finance. https://finance.yahoo.com/, (2023). Accessed: 2023-10-06.

[46] Hatcher, A. *Algebraic topology* (Cambridge Univ. Press, Cambridge, 2000).

[47] Tabaghi, P. & Dokmanić, I. Hyperbolic distance matrices, * Proceedings of the 26th ACM SIGKDD International Conference on Knowledge Discovery and Data Mining*, p. 1728–1738, (2020).

