# Supplementary material for: Integral Betti signatures of brain, climate and financial networks compared to hyperbolic, Euclidean and spherical models
Source: Sci Rep. 2025 Dec 23;16:2026. doi: 10.1038/s41598-025-31700-z (PMC12808783; doi:10.1038/s41598-025-31700-z)
Supplement: Supplementary file 1 — Supplementary Information. [file 41598_2025_31700_MOESM1_ESM.pdf]

Supplementary information
Integral Betti signatures of brain, climate and financial networks compared to hyperbolic, Euclidean and spherical models

Luigi Caputi[a], Anna Pidnebesna[a,b], Jaroslav Hlinka*[a,b]

[a] *Institute of Computer Science of the Czech Academy of Sciences,*
Pod Vodárenskou věží 271/2, 182 07 Prague, Czech Republic
[b] *National Institute of Mental Health, Topolová 748, 250 67 Klecany, Czech Republic*
[c] *Emails:* luigi.caputi@unibo.it; pidnebesna@cs.cas.cz; hlinka@cs.cas.cz *(* corresponding)*

1. Betti curves of order complexes

In this section, we briefly recall the topological pipeline introduced by Giusti *et al.* in [1]. To each symmetric matrix M, we associate specific topological features called *Betti curves*, one for each dimension $n \in \mathbb{N}$. Roughly, the Betti curve in dimension n describes the number of n-dimensional holes of a graph with adjacency matrix M, at various thresholds, as a function of the edge density. Following [1, 2], we now proceed with a detailed description of the pipeline.

1.1. The order complex

Recall that a *graph* G is a pair $G = (V, E)$ given by a finite set V, whose elements are called vertices, and a set $E \subseteq V \times V$; each element e of E, called an edge, is described by an unordered pair of distinct vertices $\{v, w\}$, which are the endpoints of e. We observe here that multiple edges between two vertices, and self-loops (i.e. edges of type $\{v, v\}$) are not allowed. We will only deal with finite graphs, which means, the set of vertices V is finite. For given graphs $G_1 = (V_1, E_1)$ and $G_2 = (V_2, E_2)$, we say that G_1 is a subgraph of G_2, and we write $G_1 \subseteq G_2$, if V_1 is a subset of V_2, and if E_1 is a subset of E_2.

Remark 1.1. Let $G = (V, E)$ be a graph and assume V to be of cardinality n. Consider a bijective function $f\colon \{1, \ldots, n\} \to V$ from the set of natural numbers between 1 and n to V. Then, we can associate to G a symmetric matrix A called an adjacency matrix of G. For indices $i, j \in \{1, \ldots, n\}$, let $A(i, j) := 1$ if, and only if, $\{f(i), f(j)\}$ is an edge of G, and 0 otherwise. As edges are given by unordered pairs of vertices, we have $A(i, j) = A(j, i)$ for all i and j in $\{1, \ldots, n\}$, and the procedure yields a symmetric matrix.

As recalled in Remark 1.1, to each graph we can associate an adjacency matrix; analogously, to each symmetric matrix with values in $\{0, 1\}$, we can associate a graph. More generally, we can consider symmetric real-valued matrices. In such case, to each $N \times N$ symmetric matrix M with distinct non-zero real-valued entries, we associate a whole family of graphs

$$\mathrm{ord}(M) := G_0 \subseteq G_1 \subseteq \cdots \subseteq G_k \ , \tag{1.1}$$

called the *order complex* of M [1, Def. 2, SI]. The construction of $\mathrm{ord}(M)$ starts from a totally disconnected graph G_0 on N vertices, and proceeds step by step by adding new edges, as indicated by the entries of M. To be more precise, the construction proceeds as follows. Let k be the number of non-trivial off-diagonal entries of M, counted without repetitions, and let $[a_1, \ldots, a_k]$ be the ordered sequence of such (distinct) real values, sorted in a decreasing order. As a first step, let G_0 be the graph on N vertices and no edges, with vertices ordered from 0 to N. Then, inductively construct G_s from G_{s-1} by adding an edge $\{i, j\}$ to G_{s-1} for indices $i, j \in \{0, \ldots, N\}$ such that $M(i, j) = a_s$. This iterative construction describes a family of graphs G_s, for $s = 0, \ldots, k$. Observe that the number k is bounded by $\binom{N}{2}$ – $\binom{N}{2}$ being the number of off-diagonal entries of M – and that each graph G_{s-1} is a subgraph of G_s, making $\mathrm{ord}(M)$ into a sequence of subgraphs of G_k.

In concrete applications, the $N \times N$ symmetric matrix M has often non-zero off-diagonal elements, and k is then equal to $\binom{N}{2}$. In the follow-up, the graphs appearing in an order complex will be always indexed by

$$\rho = \frac{s}{\binom{N}{2}} \ ,$$

the edge density of G_k.

Remark 1.2. The order complex is invariant under monotonic transformations. This property allows great flexibility in the applications and, especially in presence of non-linearity, it can be used to detect geometric signatures of structure and/or randomness [1]. Furthermore, the construction depends only on the (combinatorics of the) symmetric matrix.

In the construction, we have sorted the values of M in a decreasing order. Analogously, sorting the values in an increasing order yields another (generally distinct) family of subgraphs of G_k.

1.2. Clique complexes

Given a sequence of graphs, it is customary in Topological Data Analysis to construct a sequence of higher dimensional spaces called simplicial complexes. We recall the definition:

Definition 1.3. An *(abstract) simplicial complex* on a set V is a collection Σ of non-empty finite subsets σ of V, closed under taking subsets: if $\sigma \in \Sigma$ and $\tau \subseteq \sigma$ is non-empty, then $\tau \in \Sigma$.

The elements σ of Σ are called *simplices* and the elements of V also called vertices of Σ. The dimension $\dim(\sigma)$ of a simplex σ is given by the number of its vertices: $\dim(\sigma) := |\sigma| - 1$, where, if $\sigma = [v_1, \ldots, v_j]$ then $|\sigma| = j$. For example, a vertex $v \in V$, seen as element $\{v\}$ in Σ, has dimension 0. The dimension of a simplicial complex is the maximum dimension across its simplices. Graphs are straightforward examples of 1-dimensional simplicial complexes:

Remark 1.4. A graph $G = (V, E)$ is, in particular, a simplicial complex whose vertex set is V and the other simplices are given by the edges of G. The dimension of G, seen as a simplicial complex, is 1.

To a sequence of graphs, it is possible to associate various sequences of simplicial complexes. In this work, we consider the so-called clique complexes. Recall that a n-clique, in a graph G, is a complete sub-graph on n vertices; in particular, vertices are 1-cliques and edges are 2-cliques.

Definition 1.5. Let $G = (V, E)$ be a graph. The *clique complex* $\widetilde{G}$ associated to G is the simplicial complex on the set V, whose simplices are precisely the cliques of G.

By definition, the simplices of $\widetilde{G}$ are given by all the complete subgraphs of G. Each n-clique corresponds to a $(n-1)$-simplex of $\widetilde{G}$.

Example 1.6. If G is the complete graph on 3 vertices $\{v_0, v_1, v_2\}$, then the simplicial complex $\widetilde{G}$ consists of the vertices $\{v_0\}, \{v_1\}, \{v_2\}$, of the edges $\{v_0, v_1\}, \{v_1, v_2\}$ and $\{v_0, v_2\}$, together with the 2-simplex corresponding to the whole clique $\{v_0, v_1, v_2\}$.

Every simplicial complex can be realized geometrically. In the previous example, this geometric realization can be illustrated as follows:

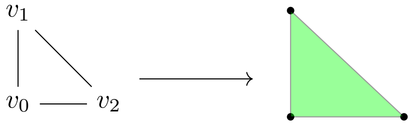

To be more precise, an n-simplex $\sigma = \{v_0, \ldots, v_n\}$ is geometrically realized as the convex hull of $n+1$ geometrically independent vectors $v_0, \ldots, v_n$ in $\mathbb{R}^{n+1}$. A 0-simplex is a point, a 1-simplex is depicted as a segment, a 2-simplex is a triangle, and so on. Then, simplices are glued together along common faces.

Example 1.7. Consider the graph G on seven vertices as described by the following picture:

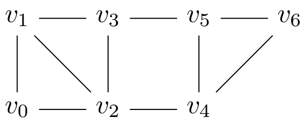

Note that, besides 1- and 2-cliques, the graph has also the 3-cliques $\{v_0, v_1, v_2\}$, $\{v_3, v_1, v_2\}$ and also $\{v_4, v_5, v_6\}$. Therefore, the associated clique complex has three 2-simplices, and these are glued together along the faces. For example, the simplices corresponding to the cliques $\{v_0, v_1, v_2\}$ and $\{v_3, v_1, v_2\}$ are glued together along the segment $\{v_1, v_2\}$. The geometric realization of the graph G is the following:

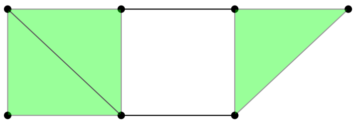

1.3. Homology of complexes

Classical topological invariants of simplicial complexes are the so-called *homology groups* [3]. For a simplicial complex Σ, the k-th homology group $\mathrm{H}_k(\Sigma)$ of Σ can be thought of as the set of k-dimensional holes of the geometric realization of Σ. To be more precise, consider the field $\mathbb{Z}_2$ with two elements (i.e. 0 and 1, with sum and product inherited from the usual sum and product of real numbers, reduced mod 2) and let $C_p(\Sigma)$ be the free $\mathbb{Z}_2$-vector space whose basis consists of the set of p-simplices of Σ. For every $p \geq 1$ we define the map

$$\partial_p \colon C_p(\Sigma) \to C_{p-1}(\Sigma), \quad \partial_p(\sigma) \coloneqq \sum_{\tau \subseteq \sigma, \tau \in C_{p-1}(\Sigma)} \tau$$

by sending a p-simplex σ to a formal sum of all its $(p-1)$-faces. The map ∂_0 is defined as the zero map. An easy computation shows that the composition $\partial_p \circ \partial_{p+1} = 0$ is the zero map – *cf.* [3] – hence, the image of ∂_{p+1} in $C_p(\Sigma)$ is contained in the kernel of ∂_p. The homology groups of the simplicial complex Σ, with $\mathbb{Z}_2$-coefficients are defined as follows:

Definition 1.8. Let Σ be a simplicial complex and $k \geq 0$ a natural number. The k-th *homology group* $\mathrm{H}_k(\Sigma)$ of Σ

$$\mathrm{H}_k(\Sigma) \coloneqq \ker(\partial_k)/\mathrm{Im}(\partial_{k+1})$$

is defined as the quotient of the kernel of the map ∂_k with the image of ∂_{k+1}.

We refrain here from giving the definition of homology groups with more general coefficients, referring to more classical texts on the subject as [3], or also the appendices of [1, 4].

Definition 1.9. Let Σ be a simplicial complex. The dimension of $\mathrm{H}_i(\Sigma)$ (over $\mathbb{Z}_2$) is called the i-th *Betti number* (over $\mathbb{Z}_2$) of Σ; the i-th Betti number is denoted by $\beta_i(\Sigma)$, or simply by β_i when the simplicial complex is clear from the context.

The group $\mathrm{H}_k(\Sigma)$ is in fact a $\mathbb{Z}_2$-vector space and its dimension over $\mathbb{Z}_2$ is well-defined. Betti numbers describe topological and geometric features of simplicial complexes. In fact, the number of k-holes of (the geometric realization of) a simplicial complex Σ corresponds to the k-th Betti number $\beta_k(\Sigma)$ associated this way to Σ. The 0-th Betti number β_0 gives the number of connected components, the 1-st Betti number β_1 the number of independent loops and the 2-nd Betti number β_2 gives the number of 2-dimensional spheres embedded in Σ.

Example 1.10. If X is a point, then its Betti numbers $\beta_n(X)$ are 0 for every $n > 0$, except for $\beta_0(X)$ which is 1. The 0-th Betti number of two points is 2 (corresponding to having 2 connected components) and 0 in higher dimensions. If we consider X to be the (geometric realization of the) simplicial complex of Example 1.7, then the Betti numbers $\beta_n(X)$ are 0 for every $n > 1$, except for $\beta_0(X)$ and $\beta_1(X)$, which are both 1 – corresponding to X having a single connected component and a cycle.

1.4. Betti curves of order complexes

We now go back to the sequence of graphs appearing in the order complex associated to a symmetric matrix. For a given order complex $\mathrm{ord}(M)$ represented as the sequence of graphs in (1.1), the pipeline explained in the previous sections yields a sequence of clique complexes

$$\widetilde{G_0} \subseteq \widetilde{G_1} \subseteq \cdots \subseteq \widetilde{G_k} \tag{1.2}$$

where also the containments $\widetilde{G}_{s-1} \subseteq \widetilde{G}_s$ are preserved. Furthermore, for each simplicial complex $\widetilde{G_j}$ in the sequence of (1.2), we can compute the Betti numbers $\beta_i(\widetilde{G_j})$, hence we can consider the sequence of i-th Betti numbers

$$\beta_i(\widetilde{G_0}), \beta_i(\widetilde{G_1}), \ldots, \beta_i(\widetilde{G_k}) \tag{1.3}$$

and, analogously, when the sequence is indexed on the edge density.

Definition 1.11. For a symmetric real-valued matrix M, the sequences of Betti numbers described in (1.3) are called the (i-th) *Betti curves* of M. The index i will be called the *Betti dimension.*

The Betti curves roughly describe the topological dynamics behind the matrix M and provide new invariants that depend only upon the relative order of the entries of the matrix [1].

2. Random, geometric and correlation matrices

We call *random* any symmetric matrix with identically independent real-valued entries, *geometric* any symmetric matrix which is obtained as the distance matrix of sample points uniformly, randomly, distributed on a manifold, and *correlation matrix* any symmetric matrix obtained as the (Pearson) correlation matrix of given time series.

The Betti curves can reliably detect both the Euclidean geometry and the random structure of symmetric matrices – see [1]; in our work we complement this observation by exploring the case of spherical and hyperbolic geometries, together with the case of correlation matrices. We review here their definitions.

2.1. Random matrices

By random matrices, we mean *random symmetric matrices with i.i.d. entries in* $[0, 1]$. Note that these matrices are adjacency matrices of ErdösRényi graphs. For an ErdösRényi graph, or the adjacency matrix of an ErdösRényi graph, we call *random clique complex* the associated clique complex – cf. Definition 1.5.

The asymptotic behaviour of the Betti curves arising from random matrices has been theoretically studied, and it is now well understood: for each $k \in \mathbb{N}$, there is an interval, depending on k, where the k-th Betti numbers of a random clique complex X are non-zero asymptotically almost surely [5]. Furthermore, the expected number $\mathbb{E}[\beta_k(X)]$ has been computed, and it has been shown that $\beta_k(X)$ satisfies a Central Limit Theorem [6]. The theoretical predicted results are asymptotic, and depend on the sizes of the input matrices as well. When the size is small (ca. 20) in fact, the behaviour of the Betti curves changes substantially [1].

2.2. Geometric matrices

We consider the metric spaces $\mathbb{R}^n$ (the Euclidean space), $\mathbb{S}^n$ (the sphere), and $\mathbb{H}^n$ (the hyperbolic space). These are the geometric models of the simply connected n-manifolds of constant curvature 0, 1 and -1, respectively. Generally speaking, the geometric properties of a manifold, endowed with different underlying metrics, can be very different.

2.2.1. Geometric (Euclidean) matrices

Euclidean geometry is the default choice in geometric and machine learning representations. The underlying manifold is the Euclidean space $\mathbb{R}^n$, endowed with the standard Euclidean distance between vectors; for $x = (x_1, \ldots, x_n)$ and $y = (y_1, \ldots, y_n)$ vectors in $\mathbb{R}^n$, the Euclidean distance

$$d_E(x, y) \coloneqq \sqrt{\sum_{i=0}^{n} (x_i - y_i)^2}$$

is the classical distance between vectors.

In our experiments, we consider N point samples uniformly independently identically distributed in $[0, 1]^d \subseteq \mathbb{R}^d$. For each such point, we get a vector $x_i \in \mathbb{R}^d$. Then, we compute the symmetric matrix E consisting of all the Eucledian distances: $E(i, j) = E(j, i) \coloneqq d_E(x_i, x_j)$. As the matrix E is symmetric, we can apply the pipeline, the associated rank matrices, and the Betti numbers by density.

2.2.2. Geometric (Spherical) matrices

In the case of spherical geometry, with curvature $+1$, we consider points sampled on a sphere $\mathbb{S}^{d-1} \subseteq \mathbb{R}^d$, endowed with the spherical distance

$$d_S(x, y) \coloneqq \arccos \langle x, y \rangle$$

where $\langle x, y \rangle \coloneqq \sum_{i=1}^{d} x_i y_i$ denotes the scalar product of x and y in $\mathbb{R}^d$. Note that this is the distance of the shortest path on the sphere (a geodesic) between x and y.

In our experiments, using a Gaussian normal distribution ($\sim \mathcal{N}(0, 1)$), we first sample N random points in $\mathbb{R}^d$ and reduce them to unit vectors – modulo their norm. We point out here that we cannot use the uniform distribution on $[0, 1]^d$ because not spherically symmetric. This process gives N unit vectors lying on the sphere $\mathbb{S}^{d-1} \subseteq \mathbb{R}^d$, hence we compute the associated matrix S consisting of the relative spherical distances. We finally take the associated rank matrix and compute the average Betti curves.

2.2.3. Geometric (Hyperbolic) matrices

Hyperbolic geometry, is a non-Euclidean geometry, in which geodesics tend to diverge (as opposed to the spherical one where geodesics tend to converge). There are many models for hyperbolic spaces with negative constant curvature. In the following, we consider the Poincaré disc model. The underlying set of the d-dimensional Poincaré disc model $\mathbb{H}^d$ is the standard open d-dimensional ball

$$B^d := \{x = (x_1, \ldots, x_d) \in \mathbb{R}^d : |x| = \sum_{i=1}^{d} x_i < 1\}$$

in $\mathbb{R}^d$, endowed with the distance

$$d_{\mathbb{H}^n}(v, w) := \operatorname{arccosh}\left(1 + 2\frac{\|v-w\|^2}{(1-\|v\|^2)(1-\|w\|^2)}\right) ,$$

where $\|x\|$ denotes the standard Euclidean norm of a vector x in $\mathbb{R}^d$. We refer to [7] for an overview of this and other models for the hyperbolic space.

In order to get random distributions of points in the hyperbolic space we use the approximation of the distribution from [8] at given radii. The random sample points have been generated in the n-dimensional ball B^n (of vectors of norm ≤ 1) – which means, in the Poincaré disc model. For a given $R \in (0, \infty)$ we have randomly generated vectors in the ball B^n_R of vectors of norm $\leq R$ by first using a uniform distribution on the sphere S^{n-1} seen as the boundary of the n-dimensional ball; then, we have used the distribution

$$\rho(r) = \frac{\sinh^{n-1} r}{\cosh^{n-1}(R-1)}$$

for getting random distances from 0. The obtained points belong to the hyperbolic space of radius R; we have then projected such points on the standard hyperbolic space $\mathbb{H}^n$ by applying the transformation

$$r \mapsto (\cosh \rho(r) - 1)/(2 + \cosh \rho(r)) .$$

Using these sample points, we have then computed the hyperbolic distances between them. These matrices are symmetric and real-valued.

2.3. Correlation matrices

The last family of matrices that we investigate is given by correlation matrices. In fact, these are among the main sources of symmetric matrices in applications. Note that correlation matrices can be seen as geometric spherical matrices. We compute the Betti numbers of correlation matrices (Pearson) on N time points, with uniform distribution in $[-1, 1]$. Matrices are then obtained as Pearson correlation of the time series.

3. Distance from data to random geometries

Figure S1, illustrates the distances from Euclidean and hyperbolic geometries to Brain, Stocks, and Climate data. The distance from each dataset to the random geometry cloud is computed as the Euclidean distance to the nearest neighbor, considering only the B1AUC. For reference, we also display the maximum distance between points within the same random geometry cloud, shown in gray (Euclidean) and black (hyperbolic). Overall, none of the three real datasets are well explained by the random geometries, except for specific parameter ranges. For the brain time series, both random distributions are similarly distant from the data for short sequences (fewer than 100 samples), after which the distance to Euclidean geometry increases slightly. For the stock data, the data exhibit a stronger hyperbolic character for the longer series (exceeding 200 points). Finally, the climate data tend to lie closer to Euclidean than hyperbolic geometry, although they are still not well described by Euclidean geometry.

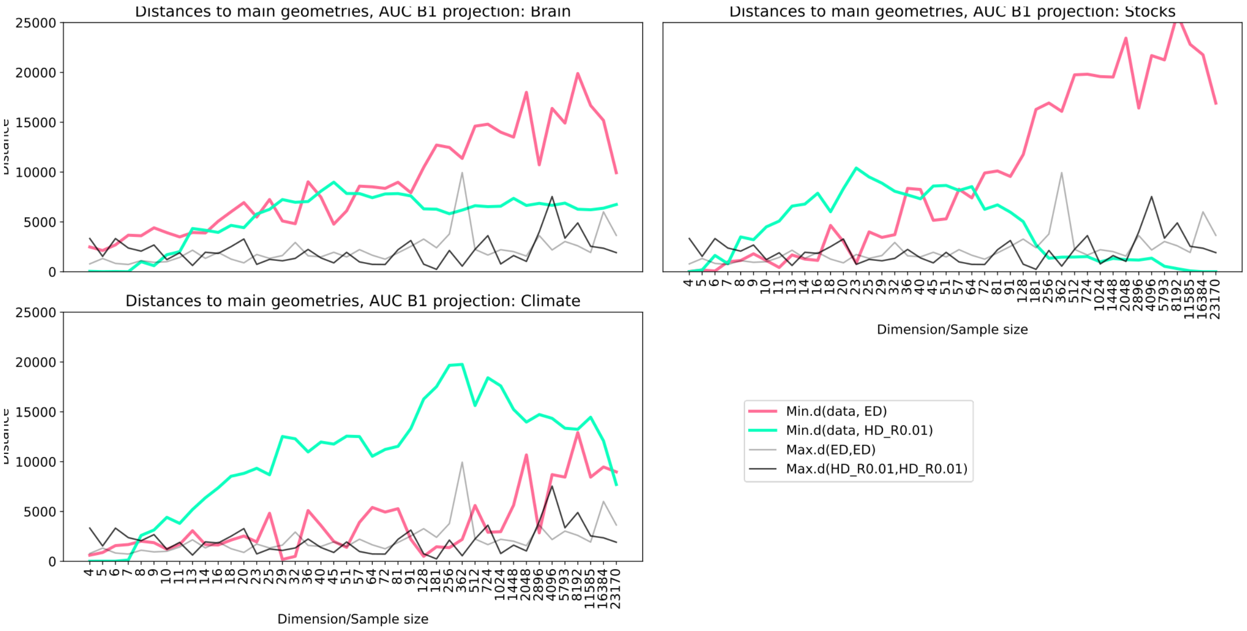

Fig. S1: Distances from the random geometries (Euclidean and hyperbolic) to the real data and distances between points inside random geometries.

4. Additional results and notes: different distance measures

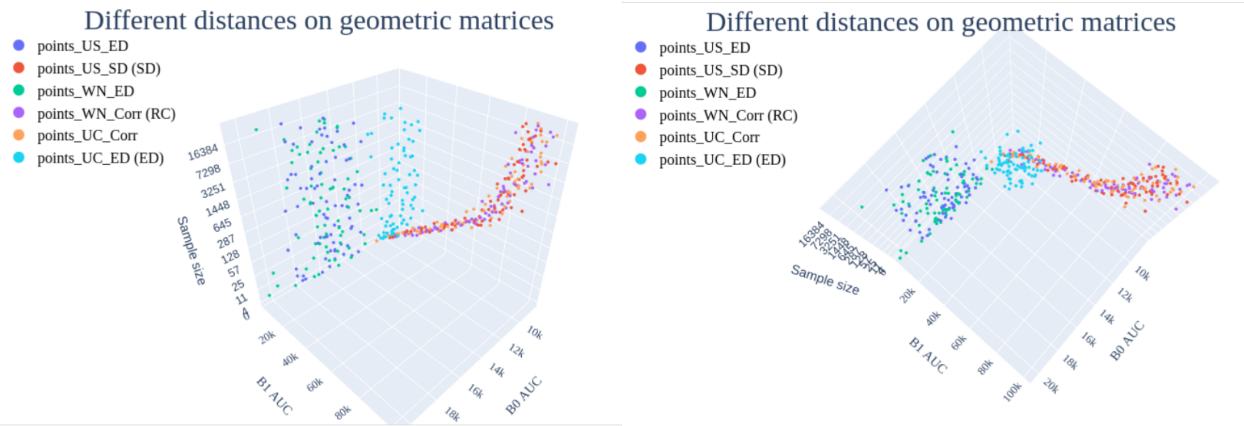

Fig. S2: Comparison of integral Betti signatures, computed for random points in the cube/sphere with different distance measures. ED denotes Euclidean distance; Corr stands for Pearson's correlation; WM means white noise (standard multivariate normal distribution); UC denotes uniformly distributed points in a hyper-cube and US - uniformly distributed points on a sphere. Observe that for correlation-based measure, the distribution of points has no effect in these particular cases. Euclidean distance matrices, computed on a white noise, have similar topology as Euclidean distance matrices, computed from the points, uniformly distributed on a sphere. However, they show more hyperbolic properties compared to the Euclidean distance matrices, computed from the points, uniformly distributed in a hyper-cube.

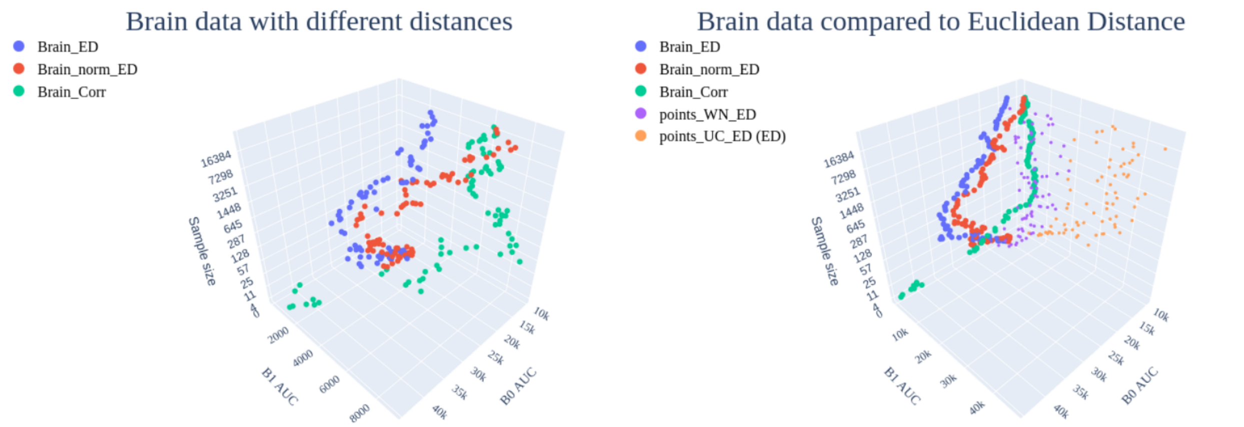

Fig. S3: Demonstration of the preprocessing influence on integral Betti signatures on the example of fMRI data. Normalization of the time series to variance 1, shifts points from hyperbolic towards euclidean geometry. Distance, computed for the data, is essential, especially for short time series, while long time series results seem to show relatively similar properties. Note also that correlation, computed from brain (fMRI) data, lies close to Euclidean distance, calculated for the white noise, however, is far from Euclidean distance of uniformly distributed points in a hyper-cube and from correlation computed on white noise. This again highlights our observation that similar properties in Betti curves should not be interpreted as necessary similarity in topology. Shortcuts: norm denotes normalization of the time series to variance 1; ED denotes Euclidean distance; Corr stands for Pearson's correlation; WM means white noise (standard multivariate normal distribution); UC denotes uniformly distributed points in a hyper-cube.

References

[1] C. Giusti, E. Pastalkova, C. Curto, and V. Itskov, "Clique topology reveals intrinsic geometric structure in neural correlations," *Proceedings of the National Academy of Sciences*, vol. 112, no. 44, pp. 13455–13460, 2015.

[2] C. Curto, J. Paik, and I. Rivin, "Betti curves of rank one symmetric matrices," in *Geometric Science of Information* (F. Nielsen and F. Barbaresco, eds.), pp. 645–655, 2021.

[3] A. Hatcher, *Algebraic topology.* Cambridge: Cambridge Univ. Press, 2000.

[4] L. Caputi, A. Pidnebesna, and J. Hlinka, "Promises and pitfalls of topological data analysis for brain connectivity analysis," *NeuroImage*, vol. 238, p. 118245, 2021.

[5] M. Kahle, "Topology of random clique complexes," *Discrete Mathematics*, vol. 309, no. 6, pp. 1658–1671, 2009.

[6] M. Kahle and E. Meckes, "Limit theorems for betti numbers of random simplicial complexes," *Homology, Homotopy and Applications*, vol. 15, no. 1, pp. 343–374, 2013.

[7] P. Tabaghi and I. Dokmanić, "Hyperbolic distance matrices," *Proceedings of the 26th ACM SIGKDD International Conference on Knowledge Discovery and Data Mining*, p. 17281738, 2020.

[8] G. Alanis-Lobato and M. Andrade, "Distance distribution between complex network nodes in hyperbolic space," *Complex Syst.*, vol. 25, 2016.
